# A systematic comparison of cardiovascular magnetic resonance and high resolution histological fibrosis quantification in a chronic porcine infarct model

**DOI:** 10.1007/s10554-017-1187-y

**Published:** 2017-06-14

**Authors:** Johannes M. I. H. Gho, René van Es, Frebus J. van Slochteren, Sanne J. Jansen of Lorkeers, Allard J. Hauer, Joep W. M. van Oorschot, Pieter A. Doevendans, Tim Leiner, Aryan Vink, Folkert W. Asselbergs, Steven A. J. Chamuleau

**Affiliations:** 1 Department of Cardiology, Division Heart and Lungs, University Medical Center Utrecht, Room E03.511, P.O. Box 85500, 3508 GA Utrecht, The Netherlands; 2 Netherlands Heart Institute, Utrecht, The Netherlands; 3 Department of Radiology, University Medical Center Utrecht, Utrecht, The Netherlands; 4 Department of Pathology, University Medical Center Utrecht, Utrecht, The Netherlands; 5 Durrer Center for Cardiogenetic Research, ICIN-Netherlands Heart Institute, Utrecht, The Netherlands; 6 Faculty of Population Health Sciences, Institute of Cardiovascular Science, University College London, London, UK

**Keywords:** Cardiovascular magnetic resonance, Fibrosis, Myocardial infarction, Late gadolinium enhancement, Strain, Mixed model

## Abstract

The noninvasive reference standard for myocardial fibrosis detection on cardiovascular magnetic resonance imaging (CMR) is late gadolinium enhancement (LGE). Currently there is no consensus on the preferred method for LGE quantification. Moreover myocardial wall thickening (WT) and strain are measures of regional deformation and function. The aim of this research was to systematically compare in vivo CMR parameters, such as LGE, WT and strain, with histological fibrosis quantification. Eight weeks after 90 min ischemia/reperfusion of the LAD artery, 16 pigs underwent in vivo Cine and LGE CMR. Histological sections from transverse heart slices were digitally analysed for fibrosis quantification. Mean fibrosis percentage of analysed sections was related to the different CMR techniques (using segmentation or feature tracking software) for each slice using a linear mixed model analysis. The full width at half maximum (FWHM) technique for quantification of LGE yielded the highest R^2^ of 60%. Cine derived myocardial WT explained 16–36% of the histological myocardial fibrosis. The peak circumferential and radial strain measured by feature tracking could explain 15 and 10% of the variance of myocardial fibrosis, respectively. The used method to systematically compare CMR image data with digital histological images is novel and feasible. Myocardial WT and strain were only modestly related with the amount of fibrosis. The fully automatic FWHM analysis technique is the preferred method to detect myocardial fibrosis.

**Electronic supplementary material:**

The online version of this article (doi:10.1007/s10554-017-1187-y) contains supplementary material, which is available to authorized users.

## Introduction

Myocardial fibrosis has been associated with heart failure and can act as a substrate for cardiac arrhythmias [1]. Following myocardial infarction (MI), loss of cardiomyocytes leads to reparative fibrosis with replacement by connective tissue. Noninvasive assessment of cardiac fibrosis is important for diagnosis, predicting prognosis and treatment planning [2]. The noninvasive reference standard for fibrosis detection is late gadolinium enhancement (LGE) on cardiovascular magnetic resonance imaging (CMR). Since there is no consensus on the LGE quantification techniques [3], a detailed comparison with the reference standard of histological analysis is important. For example, accurate fibrosis quantification can predict reversible myocardial dysfunction after revascularization [4, 5]. Thus far, mainly correlation studies have been performed with small endomyocardial biopsies, triphenyl tetrazolium chloride stained or ex vivo hearts [6–8]. Studies using whole heart slices are scarce [9], especially for focal fibrosis. While LGE provides an accurate qualitative measure of fibrosis, it requires contrast administration with potential adverse effects and does not provide a quantitative or direct measurement of cardiac collagen [2, 10]. The result of LGE differs between different imaging studies and by variable intensity threshold settings and thus relies on an adequate imaging protocol.

Functional assessment of the local myocardium is typically performed visually on cine CMR images. Quantitative assessment of local myocardial function (e.g., wall thickening) can also be performed with (semi-)automatic segmentation software packages [11, 12]. More recently, feature tracking (FT) has been introduced as a method to assess local myocardial deformation (strain) using cine images without the need for tagged CMR scans [13]. Feature tracking is relatively quick in post processing, has shown reasonable agreement with tagging CMR when looking at global strain from complete slices and might be usable with different field strengths [14–16]. However, there is debate about the agreement between strain derived from FT and tagging CMR at a segmental level, as multiple studies found poor intra- and interobserver variability for segmental strain [14, 17–19].

We have recently developed a method for high resolution systematic digital histological quantification of (diffuse and focal) cardiac fibrosis in a whole heart slice [20], which can provide a detailed reference for comparing different CMR imaging techniques. The aim of this study was to systematically analyse in vivo CMR derived parameters and high spatial resolution digital fibrosis quantification in a chronic porcine infarct model to compare different CMR techniques with myocardial fibrosis assessment. Parameters of interest are: LGE CMR, myocardial strain and wall thickening (WT). Myocardial strain and myocardial WT are respectively assessed by FT and semi-automatic segmentation on cine MRI.

## Methods

### Animal model

All in vivo experiments were conducted in accordance with the Guide for the Care and Use of Laboratory Animals prepared by the Institute of Laboratory Animal Resources. Experiments were approved (protocol no.: 2012.II.09.145) by the local Animal Experiments Committee (DEC) (Utrecht, the Netherlands).

Our protocol regarding a porcine chronic MI model has been described in detail before [21]. Eight weeks after 90 min ischemia/reperfusion of the proximal left anterior descending artery (LAD), 16 Dalland Landrace pigs (79.8 ± 5.8 kg; 6 months old; see Supplementary table 1) under continuous anesthesia underwent in vivo CMR on a clinical 3T scanner (Achieva TX, Software Release 3.2.1, Philips Healthcare, Best, the Netherlands).

### CMR

Pigs were positioned supine with a dedicated 32-channel phased-array receiver coil over the chest and scanned using a standardized protocol. For image planning scout images were obtained in short-axis and two-chamber long-axis views. ECG-gated steady-state free precession (SSFP) short-axis (from apex to base of LV) and two chamber long-axis cine images were acquired. Thirty frames were acquired per RR cycle. Cine parameters: echo time (TE)/repetition time (TR) 1.6/3.2 ms, 13 slices, slice thickness 8 mm, resolution = 2 × 2 mm, field of voxel (FOV) = 320 × 320 mm^2^, bandwidth = 1200 Hz and flip angle = 45°.

### LGE

Late gadolinium enhancement CMR was performed using an inversion recovery 3D-turbo-gradient-echo-technique 15 min after an intravenous bolus injection of 0.2 ml/kg gadobutrol (Gadovist, Bayer Healthcare, Berlin, Germany). First, a look-locker scout was performed for the optimal inversion time. Acquisition parameters for the LGE scan: inversion time (TI) = 200–270 ms, TE/TR = 1.5/4.7 ms, slice thickness = 6 mm, spatial resolution = 1.5 × 1.5 mm^2^, FOV = 300 × 300 mm^2^, flip angle = 25°, 63 TFE shots, bandwidth = 300 Hz, number of signals averaged = 2, SENSE acceleration = 2.

### CMR imaging analysis

#### Segment

**Fig. 1:**
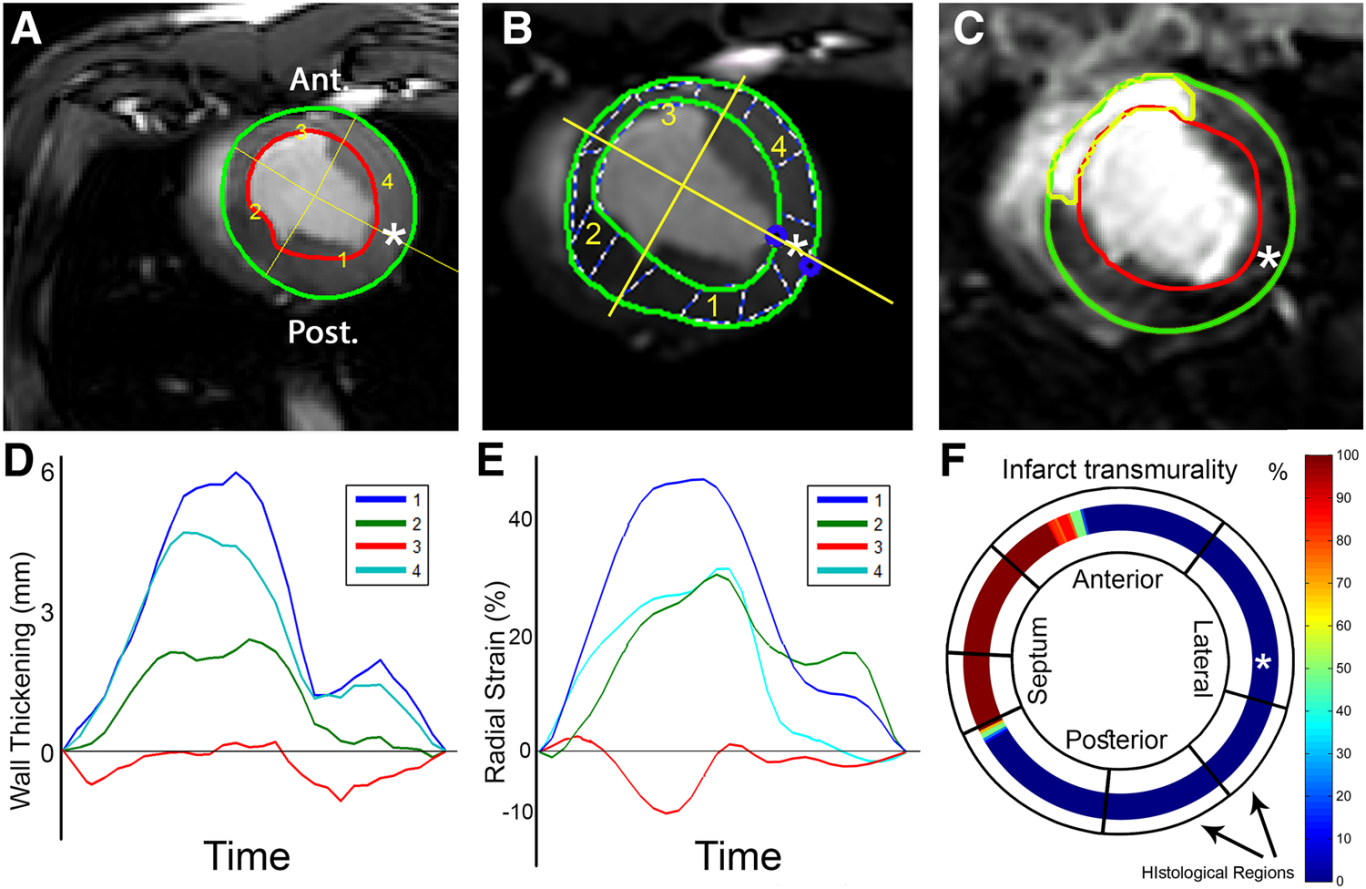
Cardiovascular magnetic resonance analysis methods. **a–c** The endo- and epicardial segmentations of the wall thickening (Segment), feature tracking (Image-Arena) and LGE (Segment) analyses respectively. The *asterisk* indicates the point in the mid-lateral wall that was used for registration between CMR and histology. **b** The *dashed lines* are used by Image-Arena as an aid for visualization of regional deformation. **c** The automatic FWHM infarct delineation is shown in C (*yellow*). **d** Resulting WT patterns of the four segments shown in **a**, the actual WT analysis used 60 sections. **e** Radial strain analysis of 48 segments averaged to result in four segments (Image-Arena); shown in **b**, the actual radial strain analysis used 48 segments. **f** The %TM data was exported in 360 segments. The digitized annotation map of the LV, as shown in Fig. 2b, is then projected onto the LGE exported data for analysis. Note: the four segments in subfigures **a, b, d** and **e** were shown as a simplified example. The actual measurements used for further analysis consisted of 60 (**a** and **d**) and 48 (**b** and **e**) segments. *FWHM* full width at half maximum, *LGE* late gadolinium enhancement, *LV* left ventricle, *%TM* fraction of transmurality, *WT* wall thickening

Offline image analysis to derive WT and LGE was performed using Segment software version v1.9 R3590 (http://segment.heiberg.se, Medviso AB, Lund, Sweden) [12]. In all datasets, one short-axis slice corresponding to the available histological slice was selected based on its location three centimetres above the apex as measured on long axis images, and used for further analysis (Fig. 1). In the short-axis cine images, LV endo- and epicardial borders were semi-automatically segmented in all time frames. The segmentation of the end-diastolic frame was copied to the corresponding LGE slice (Fig. 1c). Manual adjustment of the segmentation was performed if necessary. From the short-axis cine dataset the absolute WT (mm) per image frame of 60 LV segments was exported. The end-systolic absolute WT of each segment was used for further analysis.

#### Feature tracking

The strain analysis used in this study was performed by using the new feature tracking software Image-Arena 2D Cardiac Performance Analysis toolbox version 1.2 (TomTec Imaging Systems, Unterschleissheim, Germany). This technology tracks gray value image feature in the myocardium in the CINE images in which the end diastolic frame serves as the reference phase. The software quantifies the strain in 48 segments equally spaced over the LV myocardium. The end-diastolic endo- and epicardial contours of the selected slice were manually traced (Fig. 1b) by two authors simultaneously (R.v.E. and J.G.), based on mutual agreement. Subsequently, features along these delineations were automatically tracked with the Image-Arena software in all other frames. Correctness of the tracing was inspected manually and corrected in the frame requiring the largest adjustment, thereafter the automatic tracking was performed, these steps were repeated until the segmentation was correct in each timeframe. The mid-lateral point along the endocardial contour was selected as an anatomical reference for comparison to histology and data was exported for registration purposes. For all 48 segments, raw data containing circumferential strain (ε_cc_), radial strain (ε_rr_) and WT (endo to epi distance), was exported and used for further analysis (Fig. 1e). For the comparison with histology, the 48 strain segments are averaged to match the number of histological sections.

### Viability analysis

#### Segment

For viability analysis of short-axis LGE datasets, scar was delineated using automatic full width at half maximum (FWHM) (Fig. 1c), standard deviation (SD) from remote (2, 3 and 5SD) and manually corrected SD from remote (2, 3 and 5SD) algorithms. For the SD methods, the remote healthy myocardium of the lateral wall was selected as remote. Manual corrections of the infarct area after automatic segmentation were performed in the ‘manually corrected’ subgroups. These corrections were performed based on the expected infarct area in the LAD territory and any obvious artefacts. These regions were manually removed from the segmented scar. The fraction of area based transmurality (%TM), the infarct size fraction of the wall thickness, was analysed in 360 equal segments over the LV wall. For myocardial signal intensity (MSI) and each scar delineation method the %TM was exported for further analysis.

### Histology

**Fig. 2:**
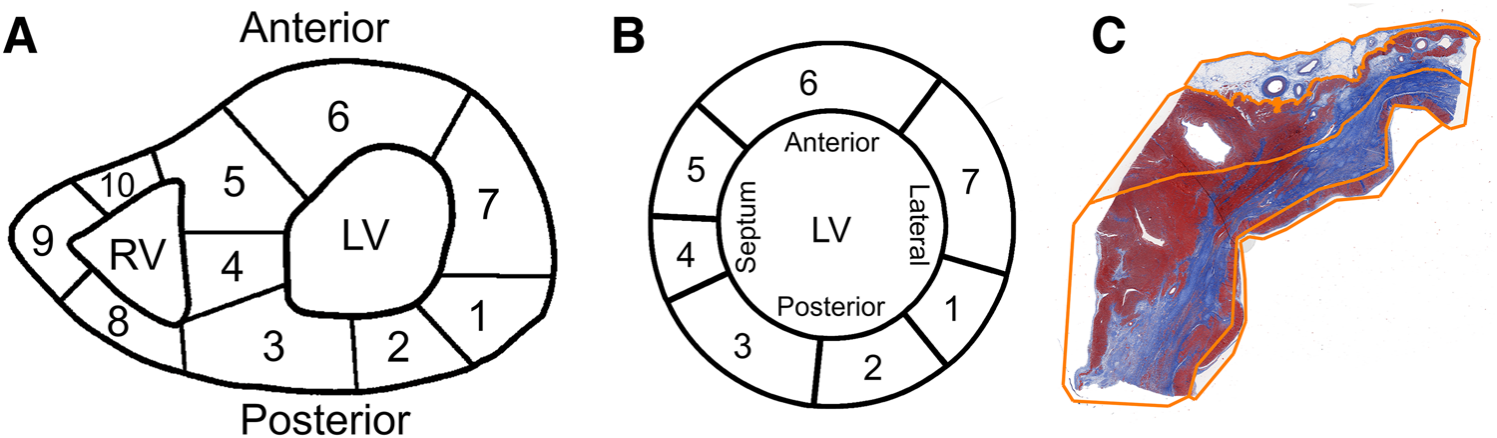
Digital histological analysis methods. **a** Example of map containing the origin of the histological sections in the heart slice of one animal. **b** The digitized left ventricular annotation map of image (**a**) **c**. The manual tracings of the four regions across the histological myocardial section, corresponding to location 5 in **a** and **b**. The *orange lines* depict the endocardial to epicardial subsegmentation

Following CMR, the animals were sacrificed by exsanguination under general anaesthesia and the hearts were excised and cut into transverse (short-axis) 1 cm thick slices from apex to base. Each third transverse slice was fixed in formalin, cut into smaller sections and an overview of the heart slice was drawn to annotate the origin of each tissue specimen (Fig. 2a). These sections were embedded in paraffin and stained with Masson’s trichrome. The slides were scanned at 20× magnification as described before [22]. Images were extracted using Aperio ImageScope v.12.0.0.5039 (Aperio, Vista, CA, USA) and resized to 10% for digital analysis.

### Histological analysis

Digital histological analysis was performed systematically as previously described, using the in-house developed open source software package Fibroquant (http://sourceforge.net/projects/fibroquant) [20]. The epicardium, defined as the outer region of fatty tissue bordered by the first row of cardiomyocytes, was excluded from further analysis. The remaining myocardium, including the compact and trabeculated region was analysed as a whole. The percentage of connective tissue (blue), cardiomyocytes (red) and adipose tissue (cells with non-stained cytoplasm; pseudo green) was digitally quantified using Fibroquant. The results were annotated to their corresponding heart region (Fig. 2a) and transformed to a standardized schematic overview (Fig. 2b).

### Registration of MRI with histology

A landmark in the mid-lateral wall (as indicated by asterisk in Fig. 1a–c, f) in all datasets was used as a reference point for registration. This point was defined as the point opposite both hinge points of the right ventricle. This mid-lateral point was selected manually in all CMR and histology datasets, subsequently the CMR dataset was rotated around the LV center point until the reference point in the CMR data was aligned with the reference point in the histology data. Thereafter the exported high detail MRI data was averaged over the regions delineated by the histological sections as shown in Fig. 1f.

### Statistical analysis

Statistics were performed using IBM SPSS Statistics (Version 20.0, IBM Corporation, Armonk, New York, United States). We compared different CMR techniques with percentages of fibrosis per section using a linear mixed model analysis. For fibrosis, the amount of residual variance (${\sigma }^{2}$) within the animals and the variance (intercept, $\tau$) between animals were calculated (null model). Subsequently, CMR parameters were added to the model (full model). R^2^ (Snijders and Bosker), was calculated as 100% minus the ratio of the full and null models (Eq. 1) [23, 24], representing the explained variance.

1 $${R^2}=1 - \frac{{\sigma _{{full}}^{2}+~{\tau _{00full}}}}{{\sigma _{{null}}^{2}+~{\tau _{00null}}}}$$

## Results

### Histology

**Fig. 3:**
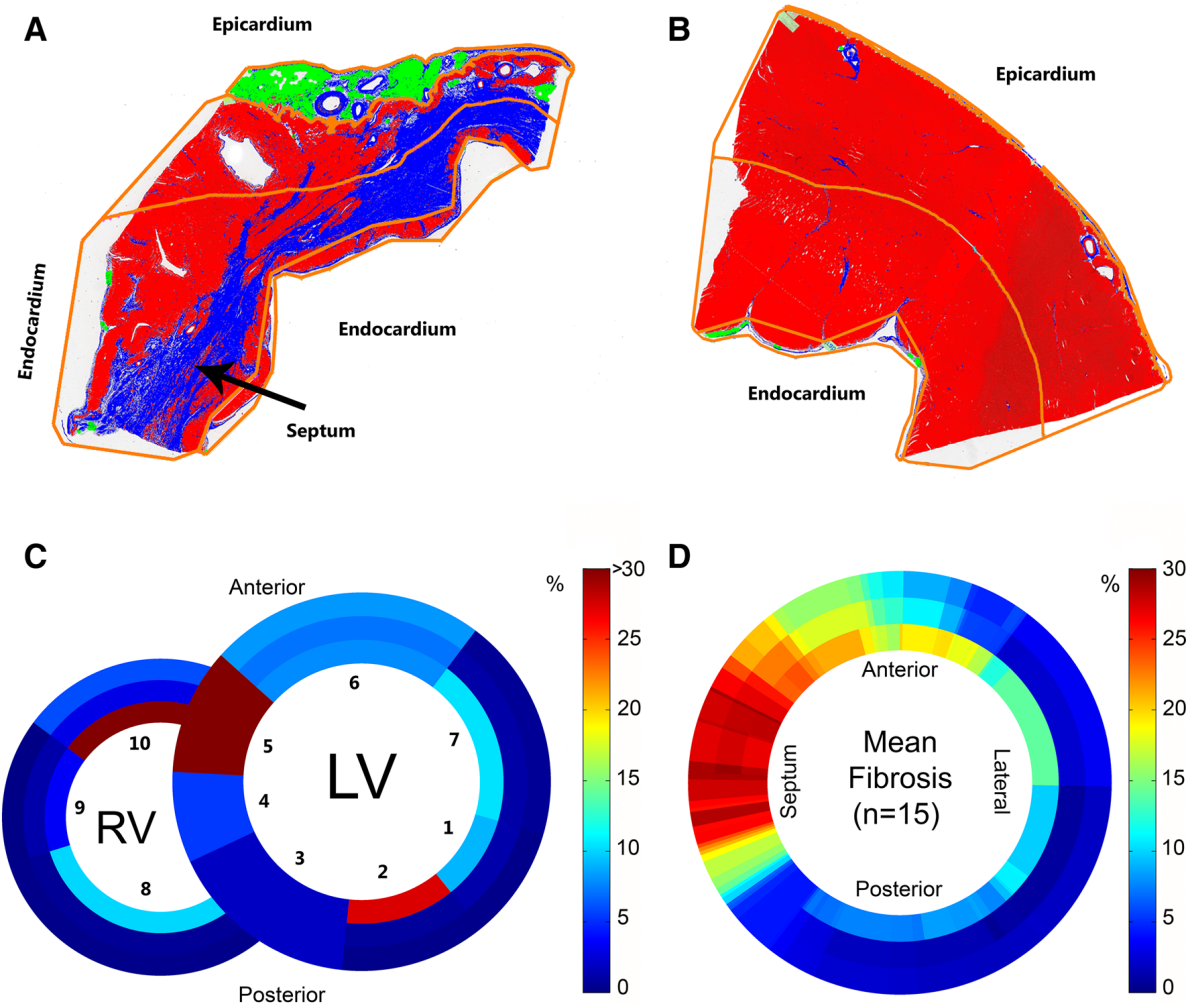
Results of histological analysis. **a, b** Results of the histological analyses of sections 5 and 7, respectively (Fig. 2a, b). Connective tissue (*blue*), cardiomyocytes (*red*) and adipose tissue (cells with non-stained cytoplasm; pseudo *green*). The *orange lines* indicate the endocardial to epicardial subsegmentation. **c, d**. The *color bar* denotes the percentage of fibrosis found based on histology. **c** Analysis of the whole heart of this animal, the numbers indicated the histological sections, 5 and 7 correspond to subfigures **a** and **b**, respectively. **d** Mean fibrosis content in the left ventricle of all animals (n = 15). *LV* left ventricle; *RV* right ventricle

A total of 116 histological sections (16 animals) were successfully stained, segmented (Fig. 2c) and analysed (see example, Fig. 3a, b). Digital maps with the annotated origins of each histological section were used to construct schematic histological overviews for each animal (see example, Fig. 3c). One animal (8 sections) was excluded from further analysis because of lack of histological fibrosis due to a sampling error (Supplementary Fig. 1), 108 sections remained. Mean fibrosis percentages (15 animals) were log transformed to reduce right-skewness and heterogeneity of variance. Myocardial fibrosis was mainly observed in the anteroseptal wall (Fig. 3d), corresponding to the LAD territory. The 8-week old LAD infarct model used in this study yielded fibrosis percentages of 57% at maximum.

### Cine CMR analysis

#### Segment

**Fig. 4:**
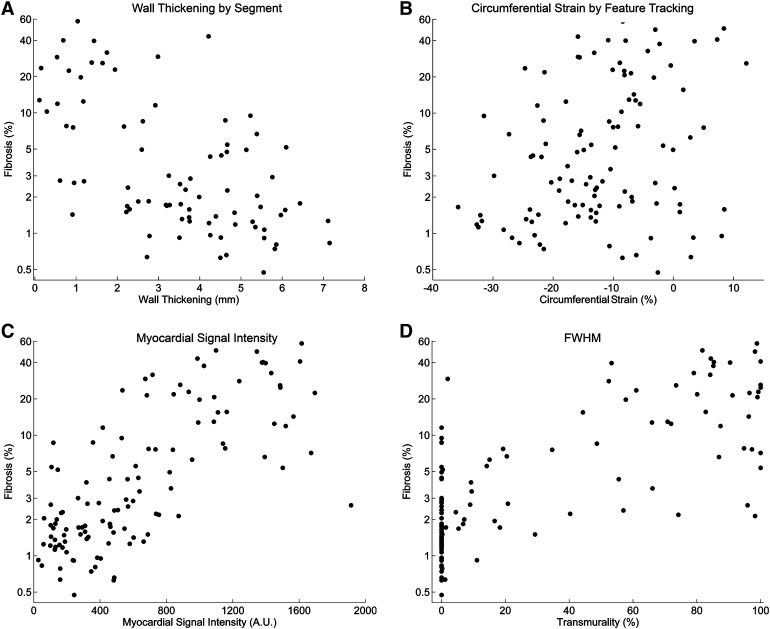
Comparisons of cardiovascular magnetic resonance parameters and fibrosis (n = 15). The *dots* represent 108 separate sections from 15 animals. R^2^ values (explained variance) are derived from a separate linear mixed model analysis. Fibrosis was compared with: **a** wall thickening results of the cine analysis (Segment); **b** circumferential strain analysis (Image-Arena); **c** myocardial signal intensity analysis (Segment); **d** LGE FWHM analysis (Segment). (A figure with a per animal linear regression line is included in the supplemental material, Fig. 2) *FWHM* full width at half maximum, *LGE* late gadolinium enhancement

**Fig. 5:**
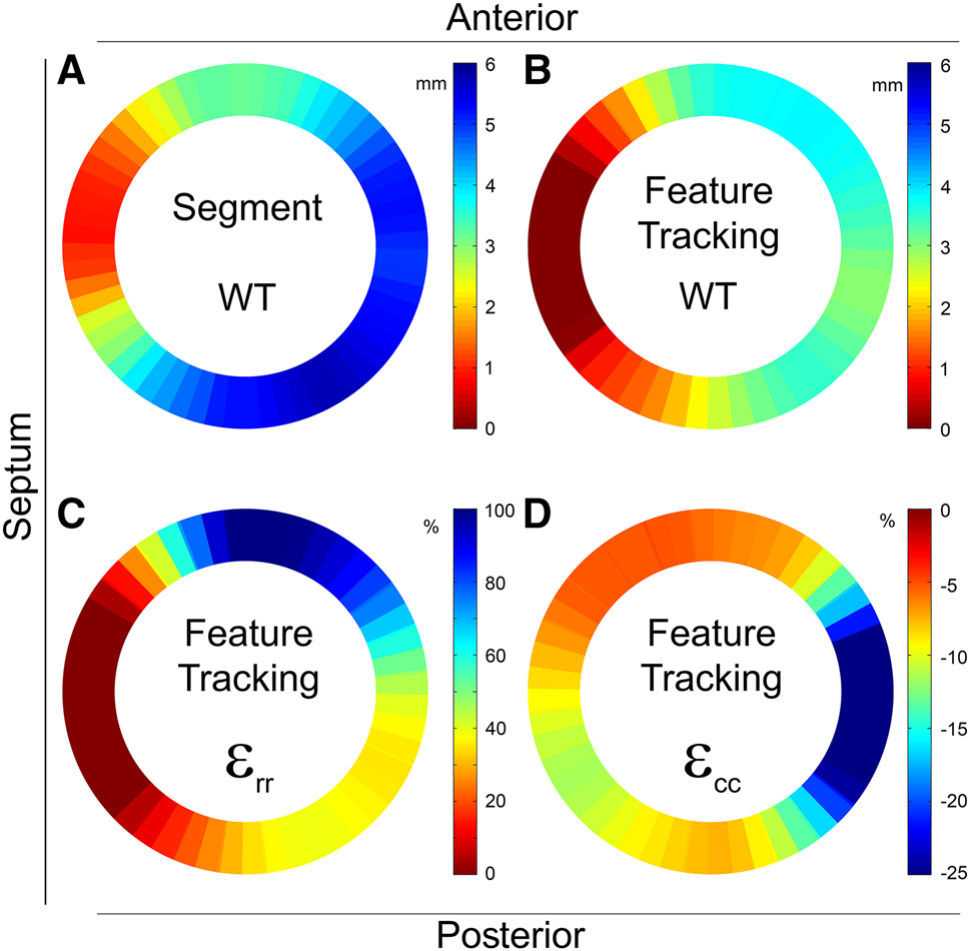
Mean wall thickening and strain analysis (n = 15). **a** The averaged results of the cine WT analysis (Segment). **b–d** The averaged results of the feature tracking analysis, the WT, circumferential (ε_cc_) and radial (ε_rr_) strain respectively (Image-Arena). *ε*_*cc*_ circumferential strain, *ε*_*rr*_ radial strain, *WT* wall thickening

Myocardial WT curves were successfully extracted for 15 animals in 60 segments. Mean end systolic absolute WT was 2.4 ± 2.4 mm (range −2.1 to 7.2 mm) (Figs. 4a, 5a). The explained variance of WT for histological myocardial fibrosis was 36% (Table 1).

**Table 1:** Comparison of wall thickening, strain and LGE with histological fibrosis

n = 15	${\sigma }_{full}^{2}$	${\tau }_{00full}$	R^2^ (%)
Cine WT (segment)	1.07	0.02	36
Cine WT (Image-Arena)	1.44	0^a^	16
Radial strain	1.54	0^a^	10
Circumferential strain	1.45	0^a^	15
2SD	0.94	0.07	41
3SD	0.82	0.11	46
5SD	0.65	0.12	55
2SD manual correction	0.66	0.03	60
3SD manual correction	0.63	0.05	60
5SD manual correction	0.65	0.07	58
FWHM	0.62	0.07	60
Myocardial signal intensity	0.68	0.14	52

The null model used in the statistical analysis containing the variance in myocardial fibrosis was expressed as ${\sigma }_{null}^{2}+ {\tau }_{00null}=1.71^*.$ The full model containing the unexplained variance in myocardial fibrosis after adding explanatory CMR parameters is expressed as ${\sigma }_{full}^{2}+ {\tau }_{full}.$ The resulting R^2^ value was calculated as 100%—the fraction of unexplained varience (Eq. 1)

^a, *^Although all convergence criteria were satisfied, the hessian matrix was not positive definite, as a consequence the residual and random intercept variance were pooled

*FWHM* full width at half maximum, *LGE* late gadolinium enhancement, *R* ^*2*^ explained variance of myocardial fibrosis, *SD* standard deviation, ${\sigma }^{2}$intra animal variance, $\tau$ inter animal variance, *WT* wall thickening

#### Feature tracking

Data of 15 animals was successfully extracted and analysed using 48 segments. After matching the data according to histological sections the mean ε_cc_, ε_rr_ and WT were −12.1 ± 12.1% (range −38.5 to 24.3%), 26.9 ± 34.7% (range −26.0 to 171.9%) and −1.6 ± 2.5 mm (range −4.6 to 7.7 mm), respectively (Figs. 4b, 5b–d). Feature tracking derived ε_cc_, ε_rr_ and WT explained 15, 10 and 16% of myocardial fibrosis, respectively (Table 1).

### Viability CMR analysis

#### Segment

**Fig. 6:**
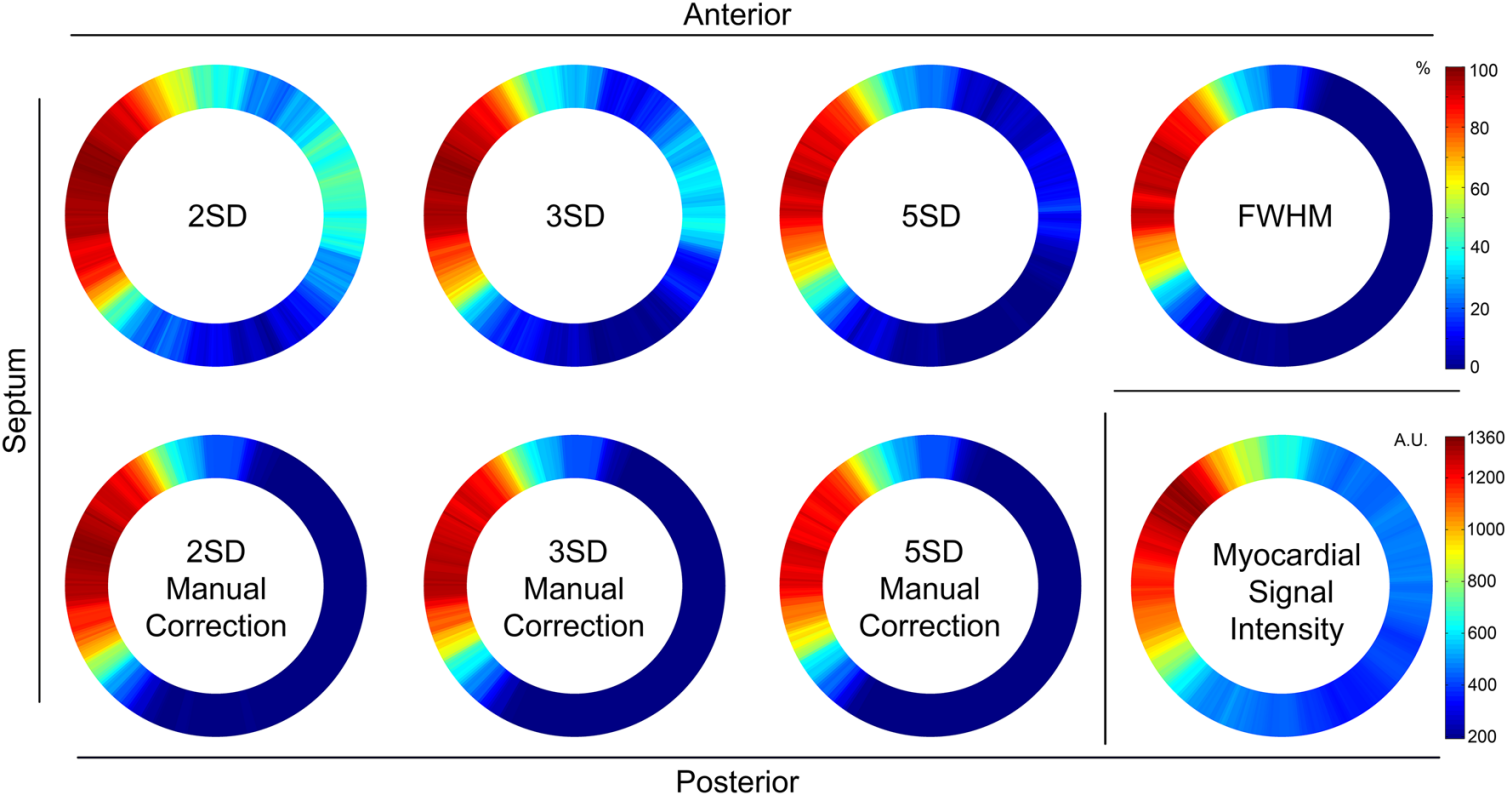
Mean late gadolinium enhancement analysis (n = 15). Averaged results of the fraction of transmurality in 360 segments for the 2, 3 and 5SD automatic methods, the 2, 3 and 5SD from remote manually corrected subgroup and the fully automatic FWHM analysis. In the bottom right corner, the averaged myocardial signal intensity is shown. *AU* arbitrary unit, *FWHM* full width at half maximum, *SD* standard deviation

The mean LGE scar transmurality and MSI (15 animals) is shown in Fig. 6. The variance between animals was small (${\tau }$ range 0–0.14) (Table 1). The automatic FWHM algorithm resulted in 60% explained variance for histological myocardial fibrosis. The manually corrected SD methods for LGE analysis yielded an explained variance between 58 and 60%. The variance within animals (σ^2^) decreased (0.94 to 0.65) with more stringent automatic segmentation methods (2–5 SD), while it remained similar in the manually corrected SD subgroup. The MSI of the LGE images, in which no additional interaction is required, explained the myocardial fibrosis for 52%.

### Scar transmurality compared to fibrosis and functional parameters

**Fig. 7:**
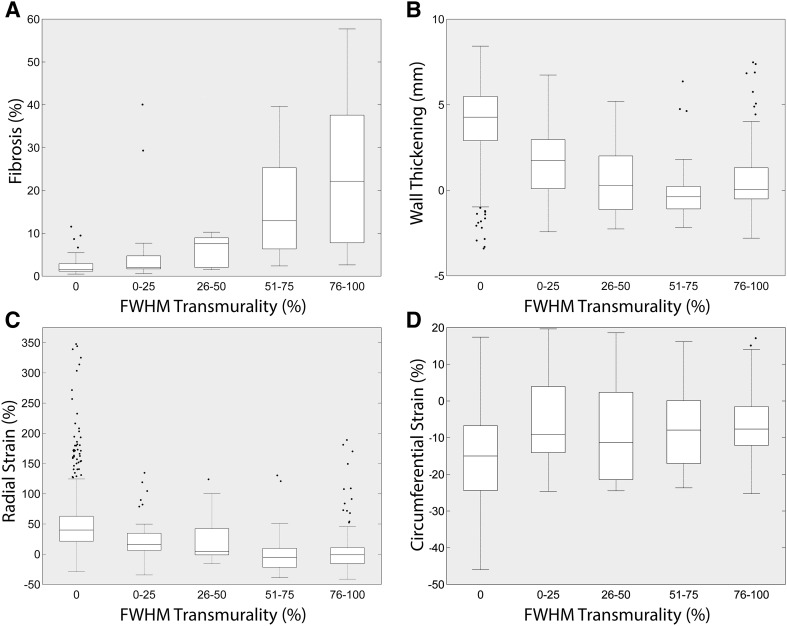
Boxplots of late gadolinium enhancement scar transmurality and fibrosis or functional parameters (n = 15). **a–d** Late gadolinium enhancement fraction of transmurality based on the FWHM algorithm divided in different subgroups (0, 0–25, 26–50, 51–75 and 76–100%) compared to fibrosis percentage, wall thickening from feature tracking, and radial or circumferential strain from FT. The *dots* represent outliers. *FT* feature tracking, *FWHM* full width at half maximum, *WT* wall thickening

In the boxplots (Fig. 7), the median fibrosis ranged from 1.4% in the segments without LGE to 22.7% in the segments with 75–100% infarct transmurality using FWHM. Median WT ranged from 4.3 to 0.0 mm and ε_cc_ and ε_rr_, ranged from −15.0 to −7.7% and 40.0 to −1.0% respectively between the infarct transmurality from 0 to 100%.

## Discussion

This study demonstrates a novel systematic method to compare different in vivo CMR techniques with high spatial resolution histological analyses in a chronic porcine infarct model. To the best of our knowledge, this is the first time such a systematic comparison has been performed and this method can be readily adapted for use with other imaging modalities such as SPECT, CT, PET and echocardiography. For LGE imaging, we found that the FWHM and the 2, 3 or 5SD from remote methods with manual correction were the best methods to quantify the amount of myocardial fibrosis. Although it is expected that measures of myocardial deformation would be affected in infarcted myocardium, myocardial WT and strain were only modestly related with the amount of myocardial fibrosis.

### Myocardial WT

The reference standard for the noninvasive assessment of cardiac anatomy and function is CMR [2]. While cine imaging is not intended for fibrosis quantification, MI is associated with a lower amount of WT in the infarct area [6]. We measured myocardial WT using two different software analysis tools, Segment and Image-Arena. Our analysis shows that WT derived from Segment performs better in explaining myocardial fibrosis compared to Image-Arena (R^2^ = 36 vs. 16%). In Segment, both the endo- and epicardial contours of the selected slice were semi-automatically traced in every frame. In Image-Arena the segmentation was performed in only one frame and the tracing in subsequent frames was automatically propagated by the software algorithm. A limitation in the FT implementation in the TomTec software is caused by the inability to perform manual adjustments in multiple frames, limiting accuracy and causing variations in the tracings. Furthermore, the slightly lower spatial resolution of the FT export compared to the Segment export (48 vs. 60 segments, respectively) may have influenced the results. Additionally, through plane motion and filtering algorithms resulting in loss of detail are likely to introduce erroneously tracked features, thereby affecting the FT results.

### Cardiac deformation

In our analysis of the LV slices, both radial and circumferential strain based on FT were poorly associated with myocardial fibrosis (R^2^ = 10 and 15% respectively). As previously described by Cowan et al., differences in strain could occur between two regions indicated as healthy by LGE [19]. From our study it can be concluded that FT based strain imaging is less applicable to identify regional myocardial fibrosis in this experimental infarct model. This might be caused by the fact that strain is not a strictly local phenomenon, but is the resulting deformation caused by the contraction of cardiomyocytes in the vicinity of the infarct. Although local myocardial deformation assessment is not a direct measurement of fibrosis, strain analysis might supply new insights in local cardiac biomechanics after ischemic injury, but rigorous clinical validation is required.

### Viability

Compared to the reference standard of fibrosis on histology, FWHM and the 2, 3 or 5SD from remote methods with manual correction were the best explanatory variables for variance in fibrosis. The FWHM technique uses half of the maximal signal intensity within the scar region as a threshold to determine the infarct area [25–27]. The used SD from remote methods delineate scar by pixels with an image intensity higher than the mean plus 2, 3 or 5SD from the mean in a non-infarcted remote region [6]. A theoretical drawback of the SD from remote technique is the manual selection of the intensity in the remote tissue, possibly limiting reproducibility. A previous clinical study showed limited reproducibility of the SD from remote techniques with manual correction and found the FWHM technique the most reproducible method [3].

In our analysis, the MSI measured from LGE images resulted in worse explanatory values for myocardial fibrosis compared to FWHM (52 vs. 60%). The between animal variance of LGE MSI compared to FWHM was higher ($\tau$ 0.14 vs. 0.07) which might be explained by MSI variation due to small differences in gadolinium injection to acquisition time. Because of the improved explanation for myocardial fibrosis and reduced manual interaction needed, FWHM would be the preferred method of choice from this study for infarct quantification on LGE CMR. In diffuse fibrosis, with less distinct LGE or remote areas and a greater influence of the partial volume effect, the FWHM method could be technically more difficult. In these situations the T1-mapping technique, in which the quantitative T1 relaxation time constant is used, might be more promising [9].

### Limitations

The final resolution of the comparison between histology and MRI is determined by the method of histological sections cut from the LV, typically 7 or 8 sections per heart slice in this study. Only one short-axis slice of the CMR images was used and the high detail MRI data was averaged to match exactly with the histology data, leading to a loss of detail, especially along the infarct border zone. For future studies, dissection of the entire heart using a (cryo)microtome could be considered to preserve the gross cardiac anatomy. The resulting high resolution images of the complete transversal heart slice can than be analyzed and subdivided to the resolution of the imaging datasets with the lowest spatial resolution to allow for a more detailed comparison with high detail imaging modalities. Hereby obviating the requirement to reduce the details of any of the datasets.

The selected mid-lateral point was used as a landmark for registration of the different modalities due to a standardized workflow for histological processing as it was used as a starting point for cutting the heart slice into smaller sections [20]. Selecting different reference points in the different datasets could have led to a rotational registration error. This type of error mainly affects the analysis in segments that contain both healthy and infarcted myocardium. For future studies we recommend to use anatomical features (e.g. RV hinge points) as landmarks for image registration, since this will further reduce the chance of a rotational registration error.

In the assessment of local cardiac deformation (e.g., WT, ε_cc_ and ε_rr_), next to the known poor intra- and interobserver variability associated with FT analysis on a segmental level, it is likely that a higher fibrosis percentage would have led to a stronger relation with worsening of functional parameters. The RV was excluded from all comparisons because the conventional analyses applied on the cine and LGE images in this study did not allow an accurate analysis of the right ventricular parameters.

### Future implications

The comparison method used in this study could be applied on data from other imaging modalities such as SPECT, PET, CT, echocardiography and other CMR sequences (e.g., T1-mapping) and can also be translated for use with a 3D model. While the proximal LAD ischemia/reperfusion model used in this study produced consequent isolated anteroseptal infarctions, it would be in the interest of external validity to study other infarct sizes and locations using this method. Improved fibrosis detection with CMR will be applicable to a broad clinical spectrum ranging from diagnostic to therapeutic outcomes, including ischemic heart disease, ablation therapy, valvular diseases, cardiomyopathies and cell therapy. For example, precise identification of the fibrotic region allows for accurate therapy guidance to the target area with the aim to ultimately improve clinical outcome for patients.

## Conclusions

In conclusion, the novel systematic method to compare high resolution in vivo CMR imaging with detailed histological fibrosis data was feasible and can readily be applied to other imaging data. Locally measured functional parameters such as WT, and measures of myocardial deformation derived from FT: radial and circumferential strain related modestly with local myocardial fibrosis, yet can be used to gain insight into local cardiac mechanics. The fully automatic FWHM algorithm applied on the reference standard LGE CMR showed to be preferred to detect myocardial fibrosis in a chronic in vivo infarct model.

## Electronic supplementary material

Below is the link to the electronic supplementary material.

Supplementary material 1 (DOCX 1062 KB)

## Abbreviations

CMR: Cardiovascular magnetic resonance imaging
ε_cc_: Circumferential strain
ε_rr_: Radial strain
FT: Feature tracking
FWHM: Full width at half maximum
LGE: Late gadolinium enhancement
LV: Left ventricle
MSI: Myocardial signal intensity
RV: Right ventricle
SD: Standard deviation
%TM: Fraction of transmurality
WT: Wall thickening

## References

[1] Weber KT, Sun Y, Bhattacharya SK, Ahokas RA, Gerling IC (2013). Myofibroblast-mediated mechanisms of pathological remodelling of the heart. Nat Rev Cardiol.

[2] Mewton N, Liu CY, Croisille P, Bluemke D, Lima JA (2011). Assessment of myocardial fibrosis with cardiovascular magnetic resonance. J Am Coll Cardiol.

[3] Flett AS, Hasleton J, Cook C (2011). Evaluation of techniques for the quantification of myocardial scar of differing etiology using cardiac magnetic resonance. JACC Cardiovasc Imaging.

[4] Kim RJ, Wu E, Rafael A (2000). The use of contrast-enhanced magnetic resonance imaging to identify reversible myocardial dysfunction. N Engl J Med.

[5] Bondarenko O, Beek AM, McCann GP, van Rossum AC (2012). Revascularization in patients with chronic ischaemic myocardial dysfunction: insights from cardiovascular magnetic resonance imaging. Eur Heart J Cardiovasc Imaging.

[6] Kim RJ, Fieno DS, Parrish TB (1999). Relationship of MRI delayed contrast enhancement to irreversible injury, infarct age, and contractile function. Circulation.

[7] Malliaras K, Smith RR, Kanazawa H (2013). Validation of contrast-enhanced magnetic resonance imaging to monitor regenerative efficacy after cell therapy in a porcine model of convalescent myocardial infarction. Circulation.

[8] Pop M, Ghugre NR, Ramanan V (2013). Quantification of fibrosis in infarcted swine hearts by ex vivo late gadolinium-enhancement and diffusion-weighted MRI methods. Phys Med Biol.

[9] Iles LM, Ellims AH, Llewellyn H (2015). Histological validation of cardiac magnetic resonance analysis of regional and diffuse interstitial myocardial fibrosis. Eur Heart J Cardiovasc Imaging.

[10] Bellin MF, Van Der Molen AJ (2008). Extracellular gadolinium-based contrast media: an overview. Eur J Radiol.

[11] Attili AK, Schuster A, Nagel E, Reiber JH, van der Geest RJ (2010). Quantification in cardiac MRI: advances in image acquisition and processing. Int J Cardiovasc Imaging.

[12] Heiberg E, Sjogren J, Ugander M, Carlsson M, Engblom H, Arheden H (2010). Design and validation of segment–freely available software for cardiovascular image analysis. BMC Med Imaging.

[13] Hor KN, Gottliebson WM, Carson C (2010). Comparison of magnetic resonance feature tracking for strain calculation with harmonic phase imaging analysis. JACC Cardiovasc Imaging.

[14] Augustine D, Lewandowski AJ, Lazdam M (2013). Global and regional left ventricular myocardial deformation measures by magnetic resonance feature tracking in healthy volunteers: comparison with tagging and relevance of gender. J Cardiovasc Magn Reson.

[15] Schuster A, Morton G, Hussain ST (2013). The intra-observer reproducibility of cardiovascular magnetic resonance myocardial feature tracking strain assessment is independent of field strength. Eur J Radiol.

[16] Khan JN, Singh A, Nazir SA, Kanagala P, Gershlick AH, McCann GP (2015). Comparison of cardiovascular magnetic resonance feature tracking and tagging for the assessment of left ventricular systolic strain in acute myocardial infarction. Eur J Radiol.

[17] Morton G, Schuster A, Jogiya R, Kutty S, Beerbaum P, Nagel E (2012). Inter-study reproducibility of cardiovascular magnetic resonance myocardial feature tracking. J Cardiovasc Magn Reson.

[18] Wu L, Germans T, Guclu A, Heymans MW, Allaart CP, van Rossum AC (2014). Feature tracking compared with tissue tagging measurements of segmental strain by cardiovascular magnetic resonance. J Cardiovasc Magn Reson.

[19] Cowan BR, Peereboom SM, Greiser A, Guehring J, Young AA (2014). Image feature determinants of global and segmental circumferential ventricular strain from cine CMR. JACC Cardiovasc Imaging.

[20] Gho JM, van Es R, Stathonikos N (2014). High resolution systematic digital histological quantification of cardiac fibrosis and adipose tissue in phospholamban p.Arg14del mutation associated cardiomyopathy. PLoS ONE.

[21] Koudstaal S, Jansen of Lorkeers S, Gho JM et al (2014) Myocardial infarction and functional outcome assessment in pigs. J Vis Exp 86:e51269 10.3791/51269 PMC4179618 24796715

[22] Huisman A, Looijen A, van den Brink SM, van Diest PJ (2010). Creation of a fully digital pathology slide archive by high-volume tissue slide scanning. Hum Pathol.

[23] Snijders TAB, Bosker RJ (2012). Multilevel Analysis: an introduction to basic and advanced multilevel modeling.

[24] LaHuis DM, Hartman MJ, Hakoyama S, Clark PC (2014). Explained variance measures for multilevel models. Organ Res Methods.

[25] Amado LC, Gerber BL, Gupta SN (2004). Accurate and objective infarct sizing by contrast-enhanced magnetic resonance imaging in a canine myocardial infarction model. J Am Coll Cardiol.

[26] Beek AM, Bondarenko O, Afsharzada F, van Rossum AC (2009). Quantification of late gadolinium enhanced CMR in viability assessment in chronic ischemic heart disease: a comparison to functional outcome. J Cardiovasc Magn Reson.

[27] Hsu LY, Natanzon A, Kellman P, Hirsch GA, Aletras AH, Arai AE (2006). Quantitative myocardial infarction on delayed enhancement MRI. Part I Animal validation of an automated feature analysis and combined thresholding infarct sizing algorithm. J Magn Reson Imaging.

