# Supplementary material for: A systematic comparison of cardiovascular magnetic resonance and high resolution histological fibrosis quantification in a chronic porcine infarct model
Source: Int J Cardiovasc Imaging. 2017 Jun 14;33(11):1797–807. doi: 10.1007/s10554-017-1187-y (PMC5682871; doi:10.1007/s10554-017-1187-y)
Supplement: Supplementary file 1 — Supplementary material 1 (DOCX 1062 KB) [file 10554_2017_1187_MOESM1_ESM.docx]

Supplemental material


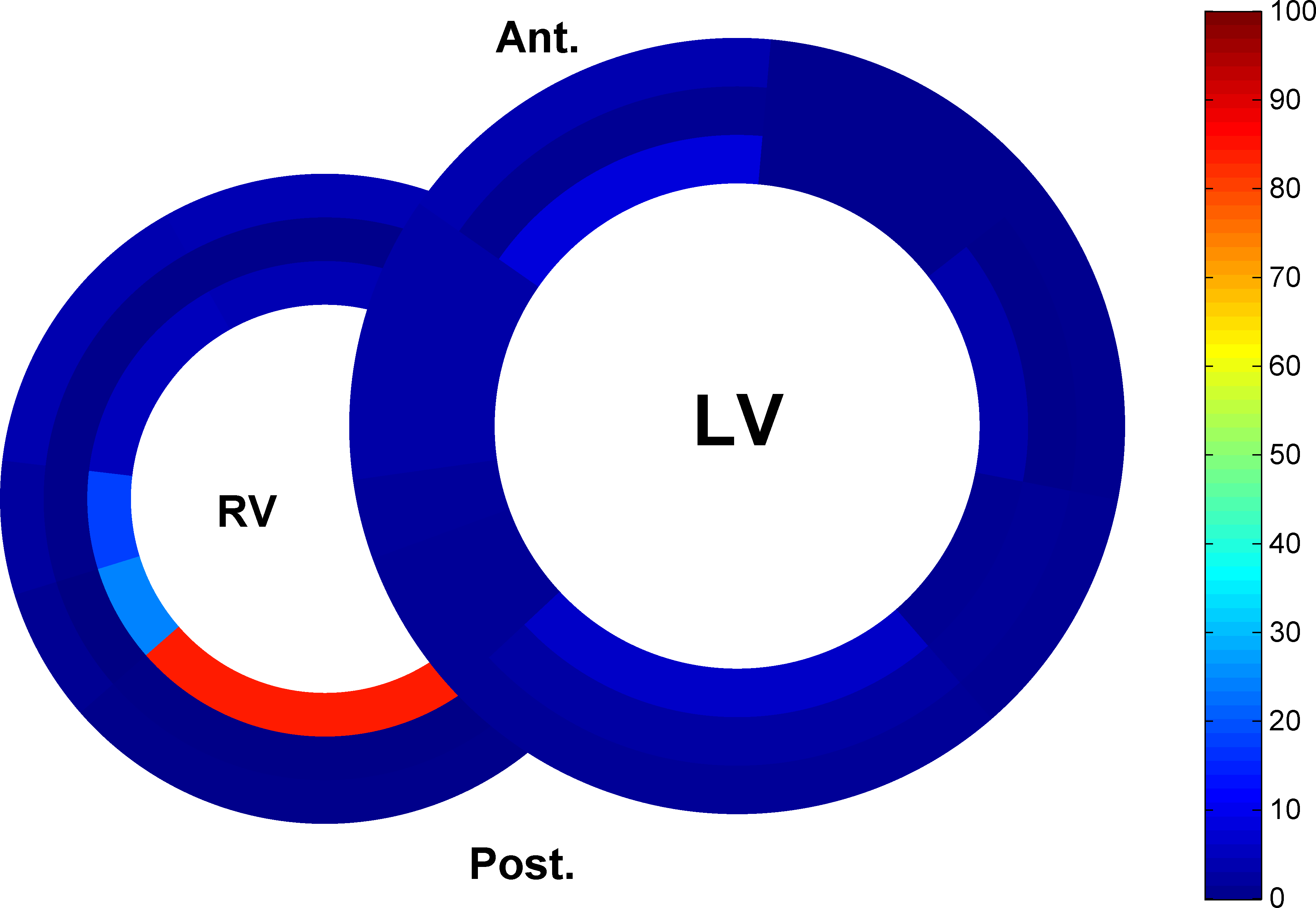


**Supplementary Figure 1** – Whole heart slice fibrosis analysis, one animal (excluded from analysis due to absence of left ventricular fibrosis). The percentage of fibrosis is shown using a color scale. Ant. = anterior; LV = left ventricle; Post. = posterior; RV = right ventricle.


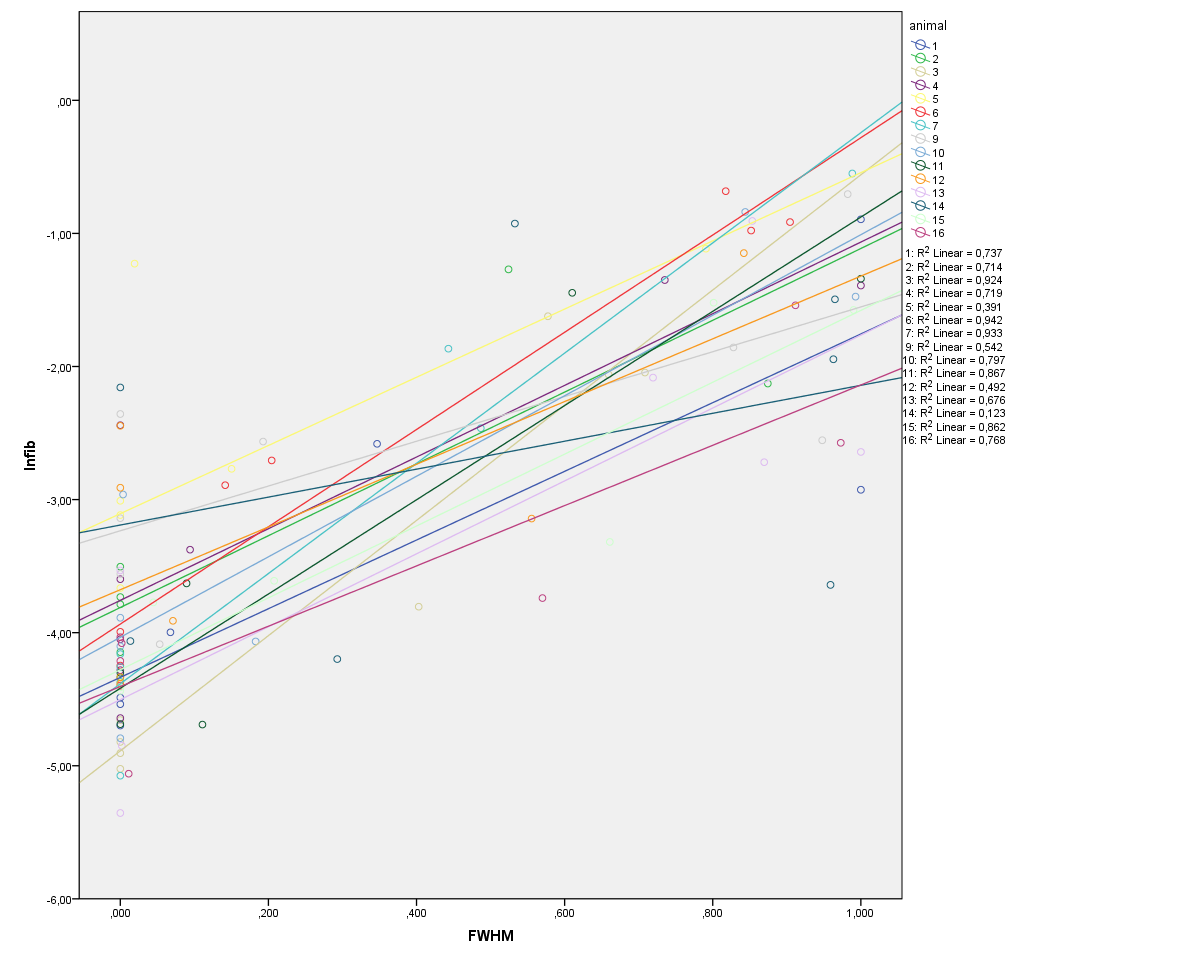


**Supplementary Figure 2 – Comparison of Full Width at Half Maximum CMR analysis and Fibrosis (n = 15).** The dots represent sections from the different animals and regression lines per animal are shown with different colors. Fibrosis was compared with LGE FWHM analysis (Segment). CMR = Cardiovascular Magnetic Resonance; FWHM = full width at half maximum; LGE = late gadolinium enhancement.

**Supplementary Table 1**

|  | Mean (±SD) |
| --- | --- |
| Left ventricular ejection fraction (%) | 39 (±6) |
| LV mass (g) | 113 (±12) |
| Infarct size (%) | 18 (±4) |
| Heart rate (bpm) | 64 (±21) |
